# Prognostic assessment and intelligent prediction system for breast reduction surgery using improved swarm intelligence optimization

**DOI:** 10.3389/fmed.2025.1653201

**Published:** 2025-09-11

**Authors:** Zhiwei Cui, Zhen Liang, Chaohua Liu, Yongjun Chen, Na Wang, Bingyang Liu, Lei Guo, Baoqiang Song

**Affiliations:** ^1^Department of Plastic Surgery, Xijing Hospital, Fourth Military Medical University, Xi’an, China; ^2^Plastic Surgery Hospital, Chinese Academy of Medical Sciences, Peking Union Medical College, Beijing, China

**Keywords:** breast reduction surgery, improved swarm intelligence optimization algorithm, AutoML, postoperative complication prediction, quality of life assessment, intelligent prediction system, clinical decision support

## Abstract

**Objective:**

This study aimed to enhance the accuracy of prognosis assessment for reduction mammaplasty by improving a swarm intelligence optimization algorithm and to develop an intelligent prediction system to support clinical decision-making.

**Methods:**

This study enrolled 224 patients who underwent reduction mammaplasty at Xijing Hospital between January 14, 2018, and February 4, 2023, and 137 patients who underwent the same procedure at Plastic Surgery Hospital between January 14, 2018, and May 1, 2020, constituting the training set. Ninety-two patients who underwent reduction mammaplasty at Plastic Surgery Hospital between May 2, 2020, and February 4, 2023, were defined as the test set. Data collection encompassed preoperative anatomical parameters, intraoperative procedural characteristics, and postoperative follow-up outcomes. Prognostic indicators included postoperative complications and the BRQS score. Guided by the Improved Secretary Bird Optimization Algorithm (ISBOA), the optimization algorithm was integrated with an AutoML framework to achieve fully automated optimization spanning from feature selection to model parameter configuration. A classification model was employed to predict the occurrence of postoperative complications, while a regression model was used to predict patient satisfaction at 1 year postoperatively.

**Results:**

The ISBOA algorithm significantly outperformed other algorithms in stability, convergence speed, and avoidance of local optima. The AutoML framework achieved an ROC-AUC of 0.9369 and a PR-AUC of 0.8856 for complication prediction (test set), and an R^2^ of 0.9165 for quality-of-life prediction (test set). SHAP analysis identified key features influencing complications and quality of life. Decision Curve Analysis (DCA) demonstrated that the AutoML model possessed high net benefit and stability across various threshold probabilities. The developed clinical decision support system could rapidly generate prediction results, aiding physicians in formulating personalized treatment plans.

**Conclusion:**

This study successfully constructed a prognosis assessment and intelligent prediction system for reduction mammaplasty based on an improved swarm intelligence optimization algorithm. The results indicate that the ISBOA algorithm exhibits significant advantages in global optimization performance and convergence efficiency. The AutoML model demonstrated excellent performance in predicting complications and assessing quality of life, with its clinical utility further validated by DCA. The developed clinical decision support system provides physicians with a convenient decision-making tool, promising to enhance the scientific rigor and efficiency of medical decision-making and offering a substantial opportunity for improving prognosis quality.

## 1 Introduction

In the field of plastic surgery, reduction mammaplasty serves as a pivotal therapeutic intervention for alleviating the physical and psychological burdens of patients with macromastia and enhancing their quality of life (1). This procedure effectively reshapes breast morphology through precise excision of redundant glandular and cutaneous tissues, significantly improving patients’ posture, body symmetry, and functional capacity for daily activities (2). However, postoperative outcomes exhibit substantial interindividual heterogeneity, particularly complication rates and the extent of quality-of-life improvement (3).

This heterogeneity arises from complex and interdependent factors, including patients’ baseline characteristics, preoperative symptom severity, and surgery-specific variables (such as unilateral/bilateral procedure, flap design pattern, volume of tissue resected per breast, nipple-to-sternal notch distance, operative duration, and postoperative hospital stay) (4). These factors profoundly influence surgical complexity, the degree of tissue trauma, and postoperative recovery trajectories (5). Consequently, systematic identification of key quantifiable prognostic factors and development of accurate predictive models are crucial for optimizing patient management and surgical outcomes (6).

Currently, the application of traditional statistical models or machine learning methods for predicting reduction mammaplasty outcomes (e.g., complications, patient satisfaction) faces significant limitations (7). A primary challenge lies in the heavy reliance on manual intervention during model development: feature selection depends on subjective expert judgment, risking omission of potentially significant variables while being time-consuming and labor-intensive; hyperparameter tuning requires extensive trial-and-error, compromising model stability and reproducibility across diverse datasets (8). Secondly, models frequently demonstrate inadequate generalization capability (9). Those trained on single-center data often fail to adequately capture variations in patient demographics, surgical techniques, and perioperative care standards across different institutions, leading to substantially degraded predictive accuracy upon external validation and severely limiting clinical utility (10).

To overcome these limitations, this study innovatively proposes an Automated Machine Learning (AutoML) framework based on a modified swarm intelligence optimization algorithm (11). This framework aims to achieve end-to-end automated optimization from feature selection to model parameter configuration. The proposed Improved Secretary Bird Optimization Algorithm (ISBOA) leverages robust global search capability, rapid convergence characteristics, and exceptional capacity for escaping local optima to efficiently navigate complex solution spaces. During feature engineering, the algorithm automatically evaluates the predictive efficacy of numerous feature combinations to identify optimal subsets; during model construction, it intelligently tunes hyperparameters, significantly reducing manual dependency while enhancing model robustness.

Critically, this study leverages multi-center clinical data (integrating datasets from Xijing Hospital and the Plastic Surgery Hospital) to effectively mitigate the constraints of single-center data. This integration not only substantially expands sample size but also incorporates geographic and inter-institutional variations in clinical practice, enabling the model to learn broader, more representative feature patterns. Consequently, the model’s generalization capability and clinical applicability are markedly enhanced. The resulting intelligent prediction system provides surgeons with scientific, personalized prognostic assessments and decision support.

The contributions and innovations of this study are summarized as follows: (1) Systematic analysis of associations between patient characteristics, preoperative status, surgical variables, and prognostic indicators (postoperative complications and BRQS-assessed quality of life); (2) Development and validation of an ISBOA-optimized AutoML framework for high-accuracy, automated prediction of complication risk and 1-year postoperative patient satisfaction; and (3) Construction of a practical clinical decision support system. This research not only provides a powerful technical tool for enhancing the precision and personalization of reduction mammaplasty but also establishes a novel research paradigm integrating swarm intelligence optimization with AutoML and multi-center big data, paving new pathways for advancing medical artificial intelligence applications.

## 2 Methods

### 2.1 Patient information

This study employed a multi-center design, integrating clinical data from Xijing Hospital and Plastic Surgery Hospital. The training cohort (*n* = 361) comprised two distinct subsets: 224 patients who underwent breast reduction mammaplasty at Xijing Hospital between January 14, 2018, and February 4, 2023, and 137 patients who underwent the same procedure at Plastic Surgery Hospital between January 14, 2018, and May 1, 2020. The test cohort consisted of an independent series of 90 consecutive breast reduction mammaplasty patients treated at Plastic Surgery Hospital from May 2, 2020, to February 4, 2023.

Sample size estimation strictly adhered to the event-driven principle for prediction model research. Based on a reported postoperative complication incidence of approximately 30% in prior literature (4) and an anticipated maximum of 10 variables for inclusion in model training, the robust modeling criterion of “events per variable (EPV) ≥ 10” mandated a minimum of 100 endpoint events. Consequently, the minimum required sample size for the training cohort was calculated as 333 patients (100/0.3 ≈ 333).

The partitioning strategy for Plastic Surgery Hospital data utilized a temporal window approach: Early data from this center (spanning 33 months) was merged with Xijing Hospital data to form the training cohort, aiming to capture potential evolution in the natural history of the condition and inherent population heterogeneity. Utilizing the center’s recent consecutive cases as an independent test cohort effectively simulated real-world clinical practice scenarios and assessed the model’s temporal generalizability on contemporary data. This design simultaneously met sample size requirements and leveraged time-truncated, independent cohorts from the same center for validation, significantly enhancing the preliminary assessment of the model’s potential for external generalizability. It also facilitated the inclusion of populations with diverse academic backgrounds.

Inclusion criteria: (1) aged between 18 and 65 years; (2) clinically assessed as meeting the criteria for breast reduction surgery; (3) signed an informed consent form preoperatively to participate in the study; and (4) received standardized rehabilitation and follow-up postoperatively for at least 1 year. Exclusion criteria: (1) previous breast cosmetic surgery; (2) other breast diseases or endocrine disorders; and (3) pregnancy or lactation. The study was approved by the Xijing Hospital’s ethics committee (No. KY20172032-F-1), with all patients providing written consent. The research adhered to the Declaration of Helsinki and relevant medical data management standards to ensure patient rights and legal compliance in data handling.

### 2.2 Data collection and follow-up methods

Data were sourced from the electronic medical records and follow-up records of breast reduction surgery patients in the hospital system from 2018 to 2023. All variables were entered independently by two individuals and cross-checked for consistency, with the database anonymized to protect patient privacy. Data were collected on preoperative anatomical parameters, intraoperative features, and postoperative follow-up outcomes. Clinical characteristics included age, body mass index (BMI), surgery type (unilateral/bilateral), flap type, unilateral tissue excision volume, nipple-to-sternum notch distance (N-SN), surgical duration, postoperative hospital stay, cardiovascular history, diabetes history, smoking history, and breast-related symptoms questionnaire (BRSQ) scores. Flap types were categorized as superomedial pedicle (SMP), inferior pedicle (IP), or superior pedicle (SP). BRSQ was measured preoperatively, validated across multiple centers, covering 13 somatic symptoms scored from 0 to 100. Prognostic indicators included postoperative complications and breast-related quality of life questionnaire (BRQS) scores. Complications such as hematoma, infection, or wound dehiscence were confirmed by the chief surgeon and classified using the Clavien-Dindo system (CDCS).

Patient follow-up strictly followed routine hospital clinical protocols, designed to systematically monitor patient recovery and assess long-term surgical outcomes. The follow-up schedule included complication surveillance within 30 days postoperatively and long-term quality of life assessment at 1 year postoperatively, enabling the construction of a comprehensive postoperative recovery database. Information on complications occurring within 30 days postoperatively was meticulously documented during outpatient reviews, ensuring timely identification and management of potential issues. Postoperative BRQL scores were collected at standardized time points (12 months ± 1 month postoperatively) via outpatient visits or mailed questionnaires to enhance patient participation and data completeness. For cases lost to follow-up, the research team conducted supplementary telephone follow-ups or utilized the last available clinical records for data compilation, minimizing the impact of missing data on study conclusions.

To ensure data quality and minimize missingness, rigorous quality control measures were implemented (detailed in Supplementary Table S1; Appendix A). Throughout data collection and follow-up, the missing rate for critical fields (including age, key surgical parameters, outcome indicators [complications], and BRQL follow-up scores) was maintained below 1%. This was achieved through stringent informed consent procedures and mandatory field requirements within the electronic medical record (EMR) system. Data for other variables underwent dual independent entry and verification, resulting in an overall low missing rate (<3%). Given the low missing rate and the absence of identifiable systematic missing patterns, a conservative and efficient data-driven missing data handling strategy was adopted: listwise deletion. Specifically, samples with missing values for a particular variable were excluded only from analytical models involving that variable. This approach avoided potential bias introduced by data imputation and was suitable for scenarios with low missing rates and non-critical independent variables. All missing data handling was completed prior to model training or validation, ensuring fair comparability between different models (note: no missing data existed for the study’s outcome variables).

### 2.3 Improvement of optimization algorithm

Optimization algorithms constitute a core component for enhancing model performance. This study employs the Secretary Bird Optimization Algorithm (SBOA) (12) for parameter optimization. As a novel swarm intelligence optimization algorithm, SBOA efficiently locates optimal solutions within complex search spaces by mimicking the survival behavior patterns of secretary bird populations (13). To achieve optimal optimization performance and enhance applicability within the subsequent machine learning framework, we implemented adaptive modifications to these algorithms, aiming to automate feature selection and hyperparameter optimization.

Building upon the original algorithm, we integrated a Sine map initialization strategy and a Cauchy mutation perturbation strategy, thereby constructing the improved secretary bird optimization algorithm (ISBOA). These strategies enhance not only the model’s predictive accuracy but also significantly improve its generalization capability. The specific implementations of the improvement strategies are as follows.

#### 2.3.1 Sine map initialization

Sine chaotic mapping replaces random initialization to enhance population diversity, as defined by equation 1:


(1) $$X_{n+1}=\frac{\mu }{4}\sin({\mathrm{\pi X}}_{n}),\;\;\mu \in [0,4],\;\;X\in [-1,1]$$

where *μ* is set to 4 to ensure maximum chaotic degree.

#### 2.3.2 Cauchy mutation perturbation strategy

To prevent premature convergence during the later iterations (*t* > 0.7 *T*), a Cauchy mutation strategy is introduced to increase global randomness. This is formulated as equation 2:


(2) $$X_{\mathrm{new}}=X_{\text{best}}+\gamma \cdot C(0,1)$$

where *C*(0, 1) represents a random number following the standard Cauchy distribution, γ=0.5×(1−t/T), *t* denotes the current iteration number, and *T* represents the total number of iterations.

Through the aforementioned strategies, the optimization algorithm not only improves the model’s predictive accuracy but also enhances its generalization capability. The performance of the improved algorithm was evaluated using the CEC2022 benchmark test functions, with comparisons made against SBOA, the Genetic Algorithm (GA) (14), Harris Hawks Optimization (HHO) (15), and the Whale Optimization Algorithm (WOA) (16). Upon identifying the optimal optimization algorithm, it will be utilized for feature selection and model tuning. During the feature selection stage, the swarm intelligence algorithm enables automated dimensionality reduction of the feature space, balancing prediction accuracy against model complexity. In the model tuning stage, the swarm intelligence algorithm efficiently searches for the optimal hyperparameter combination for the machine learning model.

### 2.4 Construction of predictive model

**Figure 1:**
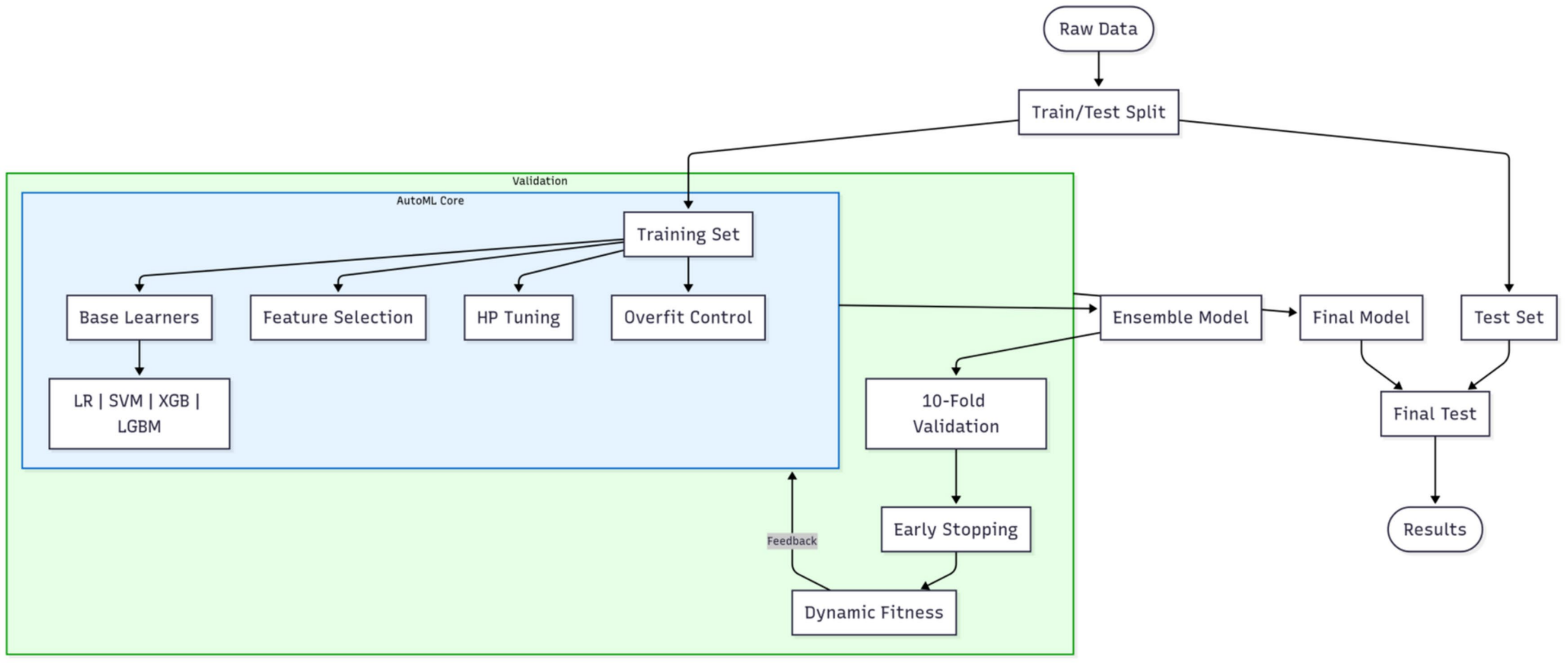
AutoML training workflow. The blue boxes represent the core AutoML process, while the outer green box indicates the integration of cross-validation.

Model construction constitutes a critical step for achieving precise prediction. This study innovatively integrates optimization algorithms with an Automated Machine Learning (AutoML) framework to realize end-to-end automated optimization from feature selection to model parameter configuration. The proposed optimization algorithm-based AutoML framework deeply incorporates a quadruple synergistic mechanism encompassing base learner selection, feature screening, hyperparameter optimization, and overfitting prevention. The AutoML training workflow is illustrated in Figure 1.

To ensure rigorous evaluation, the original dataset was partitioned via stratified random sampling into a training set and a reserved independent test set at the outset of the experiment. All subsequent procedures—including feature selection, model configuration optimization, overfitting prevention strategy deployment, and cross-validation assessment—were strictly confined to the training set. This framework unifies four decision spaces into a composite solution vector: the base learner type is a discrete variable (k: 1 = Logistic Regression (LR), 2 = Support Vector Machine (SVM), 3 = XGBoost, 4 = LightGBM); feature selection employs 0/1 binary encoding; the hyperparameter space dynamically adapts to the selected base learner; an overfitting prevention module introduces the regularization strength coefficient *λ*∈[0, 1], dropout rate *δ*∈[0, 0.5], and a data augmentation flag (a: 0 = off, 1 = on). The entire optimization process is driven by a swarm intelligence algorithm, with each iteration comprising the following core operations. First, the candidate base learner type is determined based on the k-value within the solution vector; the feature subset is selected, and corresponding overfitting prevention measures are activated. Traditional models (LR/SVM) incorporate an elastic net regularization term (strength controlled by *λ*), as defined by equation 3:


(3) $$\min_{w}[\frac{1}{N}\sum_{i=1}^{N}L(y_{i},f(X_{i})+\lambda (a{\left\|w\right\|}_{1}+(1-a){\left\|w\right\|}_{2}^{2})]$$

Tree-based models (XGBoost/LightGBM) have explicit regularization constraints applied as defined by equation 4:


(4) L(ϕ)=∑il(y^i,yi)+∑k(γT+λ2wkA2)

Simultaneously, dropout is implemented in training batches according to the *δ*-value, and the Mixup linear interpolation data augmentation strategy is activated based on the a-value. The configured model instance is then evaluated within the training set using an enhanced 10-fold cross-validation system—each fold incorporates an early stopping mechanism (training halts if validation loss fails to decrease for 5 consecutive epochs)—forming a four-dimensional synergistic feedback loop encompassing “model architecture – feature representation – parameter configuration – generalization control.” The co-optimization objective is defined by a dynamically weighted fitness function, as defined by equation 5:


(5) $$\begin{array}{l} F(x,t)=w_{1}(t)\cdot \mathrm{ACC}-w_{2}(t)\cdot (\delta +\frac{\lambda }{5})+\\ w_{3}(t)\cdot (\frac{FS_{\text{rate}}}{e^{t/50}})-\\ w_{4}(t)\cdot |\mathrm{Los}s_{\text{train}-}\mathrm{Los}s_{\text{valid}}| \end{array}$$

This function innovatively integrates four key dimensions: model accuracy (ACC), feature sparsity (*FS*_rate_), computational time cost (exponential decay term), and generalization capability difference factor (training/validation loss gap). The weighting coefficients *ω*₁ ~ ω₄ are dynamically adjusted across iteration rounds t: initial stages prioritize accuracy improvement (ω₁ = 0.6, ω₄ = 0.1), mid-stages balance accuracy and generalization (ω₁:ω₄ → 1:1), while final stages emphasize model compactness and stability (ω₂ + ω₃ account for 65%, ω₄ ≥ 0.3). Both traditional machine learning models (LR and SVM) and ensemble learning models (XGBoost and LightGBM) are included, with the effectiveness of the overfitting prevention mechanisms validated through parameter comparisons of regularization strength *λ* and dropout rate *δ*.

Two model types were employed to address distinct prediction needs. All analyzes were conducted in MATLAB 2024b. Classification models predict the occurrence of postoperative complications. Accurate classification provides clinicians with decision support for early intervention, thereby improving patients’ postoperative recovery quality. Regression models predict patient satisfaction at the 1-year postoperative mark. Predicting this long-term efficacy indicator is crucial for evaluating patient quality of life and surgical outcomes, aiding physicians in preoperative counseling and patient education.

### 2.5 Model evaluation and extended analysis

This section includes evaluation metrics, interpretability analysis, and the development of an intelligent prediction system.

For classification models, performance metrics included accuracy (ACC), sensitivity (SEN), specificity (SPE), the F1-score, the area under the receiver operating characteristic curve (AUC-ROC), and the area under the precision-recall curve (PR-AUC) were employed to evaluate the performance of classification models. Calibration curves, in conjunction with the Brier score (lower values indicate better accuracy), were used to assess probabilistic prediction accuracy. The DeLong test was applied to compare the statistical significance of differences in AUC-ROC between different models (17). For regression models, performance was evaluated using the coefficient of determination (*R*^2^), mean squared error (MSE), root mean square error (RMSE), mean absolute error (MAE), and mean absolute percentage error (MAPE).

SHapley Additive exPlanations (SHAP) was used to explore the interpretability of predictive models (18). Based on the Shapley value concept from game theory, SHAP assigns an importance value to each feature, quantifying its contribution to model predictions. Summary and importance plots were generated to visually present model interpretability.

Using MATLAB 2024a’s App Designer, a clinical decision-support software was developed. Integrating constructed predictive models, this user-friendly tool aids clinicians in assessing patient conditions and devising personalized treatment plans. With a simple interface, users input clinical data to obtain real-time model predictions and treatment recommendations.

## 3 Results

### 3.1 Comparison of baseline data

Table 1 presents the preoperative clinical characteristics of patients in the training and test sets. Results show no significant differences in age, sex, BMI, surgical type, or flap type between the two groups (*p* > 0.05). This indicates good comparability in basic clinical features, providing a reliable foundation for subsequent model evaluation.

**Table 1:** Comparability analysis of training and test sets.

Variable	Training set (*n* = 361)	Test set (*n* = 90)	Statistic	*p*-value
Age (years, mean ± SD)	48.52 ± 9.36	47.83 ± 8.97	0.631	0.529
BMI (kg/m^2^, mean ± SD)	28.50 ± 5.02	28.24 ± 4.95	0.441	0.660
Unilateral/bilateral (bilateral, *n*, %)	289 (80.06%)	64 (71.11%)	3.389	0.066
Flap Type (*n*, %)			1.396	0.498
Superomedial pedicle (SMP)	222 (63.16%)	51 (56.67%)		
Inferior pedicle (IP)	99 (27.42%)	30 (33.33%)		
Superior pedicle (SP)	34 (9.42%)	9 (10.00%)		
Unilateral resection weight ≥650 g (*n*, %)	139 (38.50%)	29 (32.22%)	1.216	0.270
N-SN (cm, mean ± SD)	32.12 ± 7.38	33.11 ± 8.65	1.099	0.273
Operation time (minutes, mean ± SD)	142.63 ± 32.74	140.85 ± 31.56	0.465	0.642
Postoperative hospital stay (days, mean ± SD)	2.81 ± 1.22	2.72 ± 1.16	0.632	0.528
Cardiovascular disease (*n*, %)	85 (23.55%)	16 (17.78%)	1.379	0.240
Diabetes (*n*, %)	21 (5.82%)	8 (8.89%)	1.130	0.288
Smoking (*n*, %)	23 (6.37%)	9 (10.00%)	1.439	0.230
Preoperative BRSQ score (points, mean ± SD)	57.38 ± 15.76	58.33 ± 16.29	0.508	0.612

BMI, body mass index; N-SN, nipple-to-sternum notch distance.

### 3.2 Assessment of optimization algorithms

**Figure 2:**
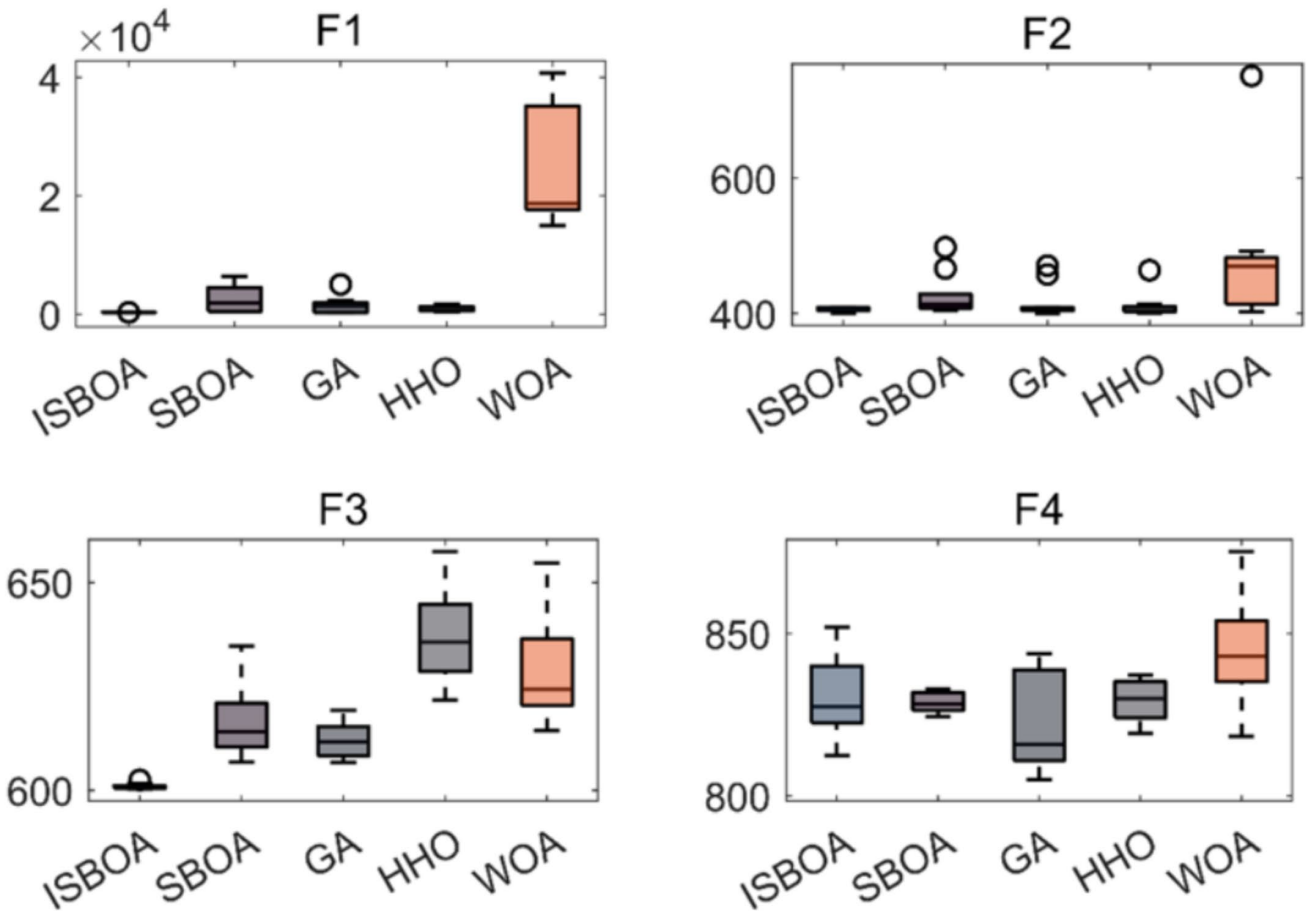
Comparison of optimization performance of swarm intelligence algorithms. Box plots of optimization results from 30 independent runs on CEC2022 test functions, showing the stability and robustness of each algorithm. ISBOA, improved secretary bird optimization algorithm; SBOA, secretary bird optimization algorithm; GA, genetic algorithm; HHO, Harris hawks optimization; WOA, whale optimization algorithm. The horizontal axis shows the name of the optimization algorithm, and the vertical axis shows the function value. These test functions are used to test the performance of optimization algorithms in different situations.

**Figure 3:**
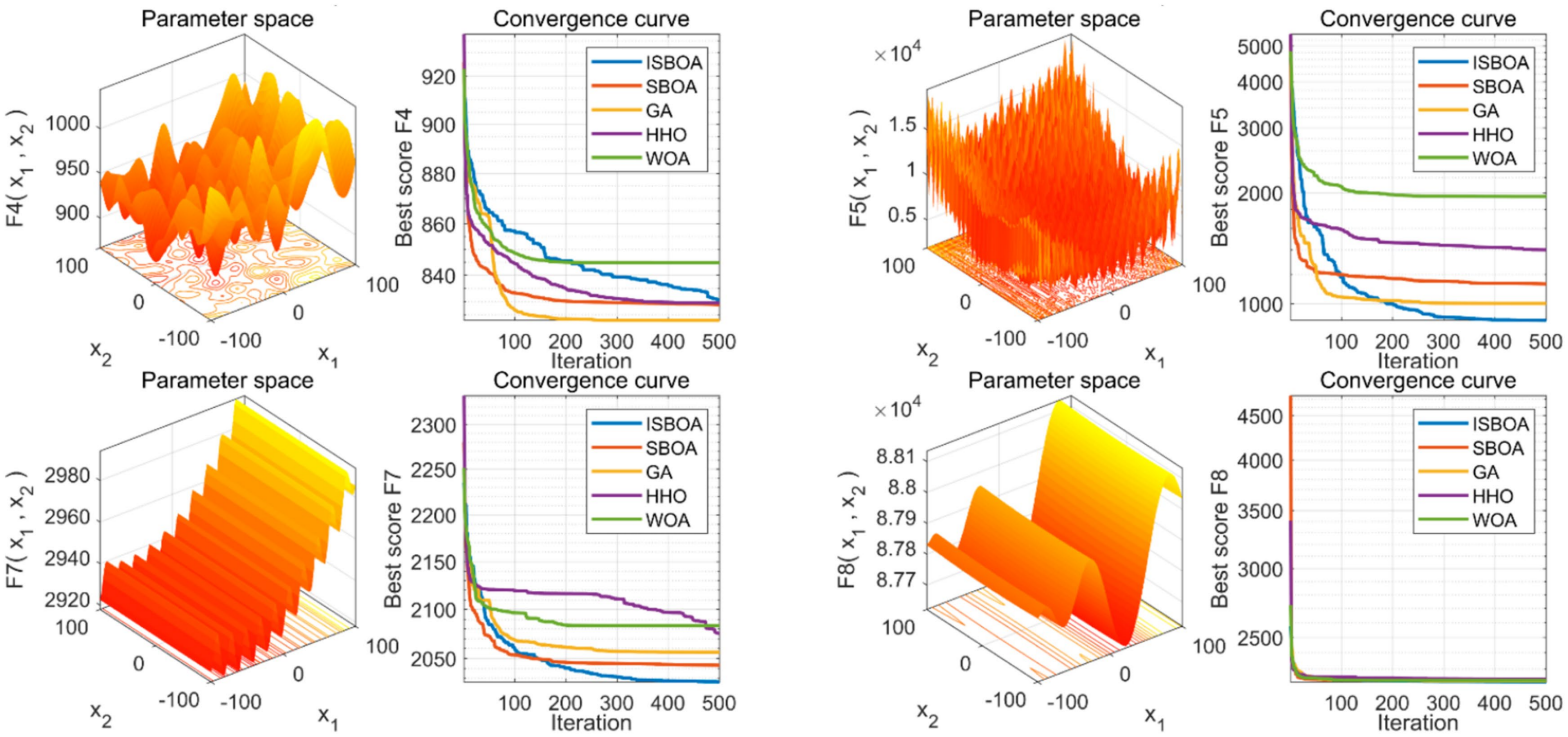
Comparison of convergence performance of swarm intelligence algorithms. Convergence curves of each algorithm during optimization, reflecting their convergence speed and ability to avoid local optima. ISBOA, improved secretary bird optimization algorithm; SBOA, secretary bird optimization algorithm; GA, genetic algorithm; HHO, Harris hawks optimization; WOA, whale optimization algorithm. In the 3D graph, the x-axis and y-axis stand for an input parameter, defining a variable dimension in the optimization problem. The z-axis shows the objective function’s output given the x and y variables, reflecting the function’s performance under different parameter combinations and serving as the optimization target. In the 2D convergence curve graph, the x-axis represents the number of iterations, and the y-axis represents the objective function value of the optimal solution found in each iteration.

The performance of our proposed ISBOA was compared with SBOA, GA, HHO, and WOA algorithms on CEC2022’s 12 benchmark functions to verify its optimization capability. Test functions had a variable dimension of 10, a population size of 30, and a maximum of 500 iterations, with 30 independent runs for statistical reliability. Box plots of the 30 runs were drawn to assess the optimization stability of each algorithm. Results show that ISBOA outperformed the original SBOA and other algorithms in most test functions (Figure 2). Convergence curve analysis further indicates that ISBOA has faster convergence and a lower risk of falling into local optima during iteration (Figure 3). Thus, ISBOA shows significant advantages in global optimization and convergence efficiency, making it suitable for subsequent feature selection and model tuning.

### 3.3 Evaluation of classification model performance

This study enrolled a total of 451 cases, among which 150 patients developed postoperative complications. In the training set comprising 361 patients, postoperative complications occurred in 32.96% (119 cases), while in the test set of 90 patients, the incidence was 34.44% (31 cases). Consequently, no statistically significant difference in postoperative complications was observed between the training and test sets (*p* > 0.05), indicating comparability.

**Figure 4:**
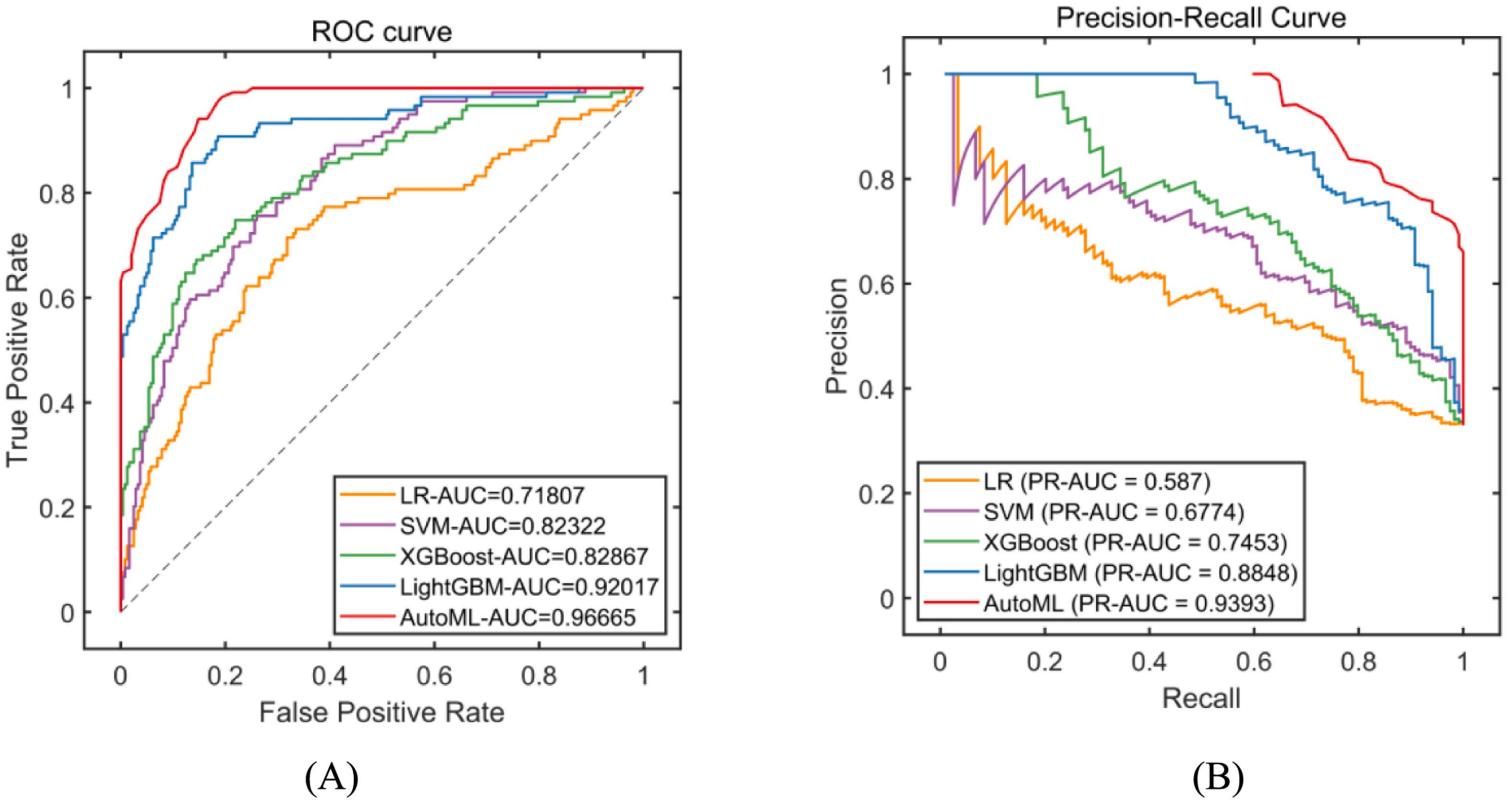
Evaluation of classification model performance-training set. **(A)** ROC curve for training set; **(B)** PR curve for training set. The left subplot is the ROC curve. The horizontal axis represents the False Positive Rate (FPR), which is the ratio of negative instances incorrectly classified as positive to the total number of actual negative instances. The vertical axis represents the True Positive Rate (TPR), also known as recall, which is the ratio of positive instances correctly classified to the total number of actual positive instances. In the legend, LR stands for Logistic Regression, SVM for Support Vector Machine, XGBoost for eXtreme Gradient Boosting, LightGBM for Light Gradient Boosting Machine, and AutoML for Automated Machine Learning. The right subplot is the Precision-Recall Curve. The horizontal axis represents Recall (same definition as TPR in the ROC curve). The vertical axis represents Precision, which is the ratio of positive instances correctly classified to the total number of instances classified as positive. The legend includes the same algorithms as the ROC curve. PR-AUC stands for Precision-Recall Area Under the Curve.

**Figure 5:**
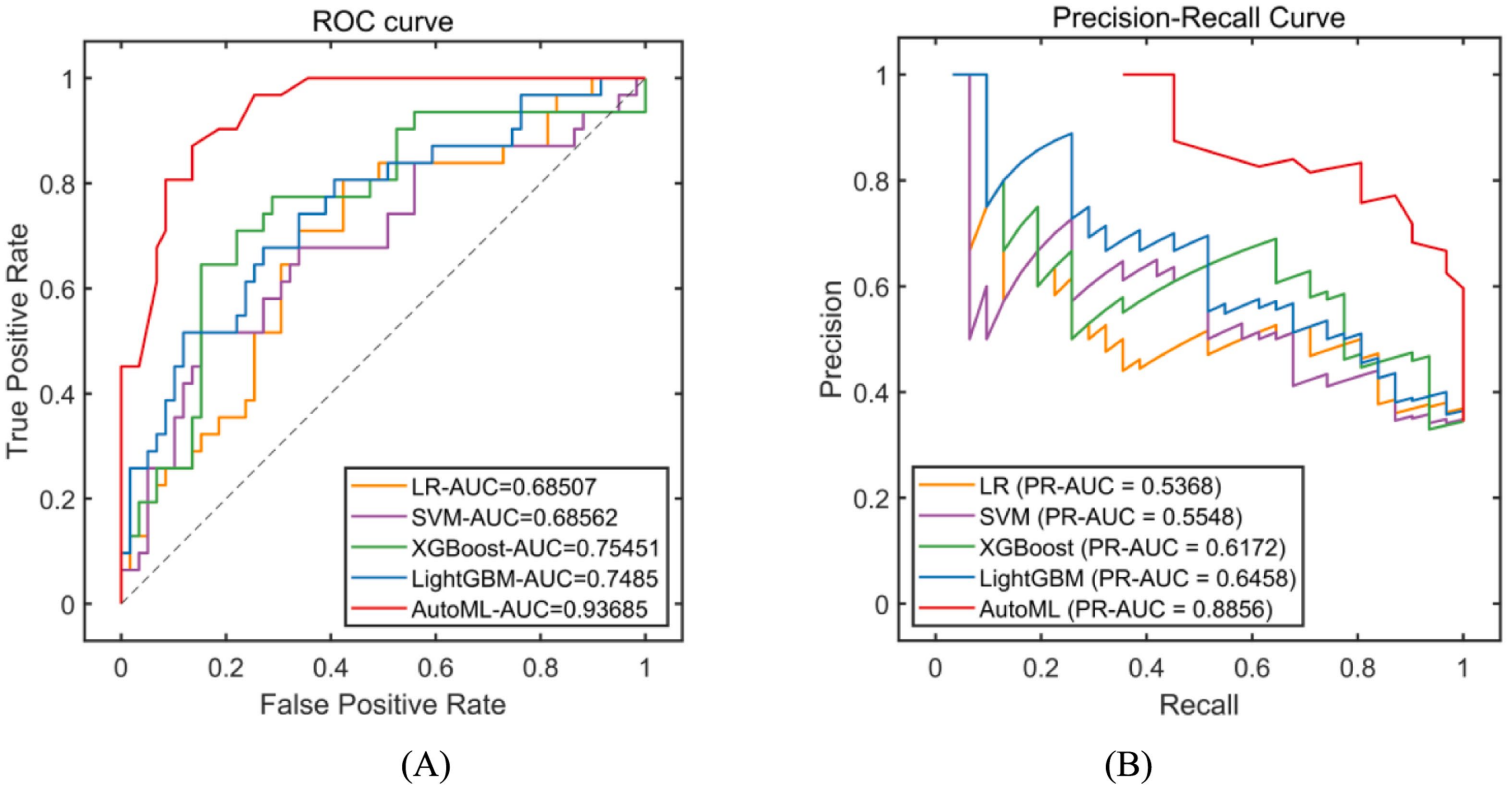
Evaluation of classification model performance-test set. **(A)** ROC curve for internal test set; **(B)** PR curve for internal test set. The explanation of the coordinate axes and the full names of their abbreviations can be seen in Figure 4.

Classification models were employed to predict complication occurrence. In the training set, 5-fold cross-validation revealed that AutoML achieved the optimal predictive performance, with ROC-AUC of 0.9667 and PR-AUC of 0.9393, significantly outperforming other models (DeLong test *p* < 0.05 for all), as detailed in Table 2 and Figure 4. In the test set, AutoML consistently demonstrated superior predictive performance, yielding ROC-AUC of 0.9369 and PR-AUC of 0.8856, significantly exceeding other models (DeLong test *p* < 0.05 for all), as presented in Table 3 and Figure 5. Key features identified by the classification models included: unilateral resection mass ≥ 650 g, BMI, bilateral surgery, age, and flap type.

**Table 2:** Evaluation of classification model performance-training set.

Model	PRE	SEN	SPE	ACC	F1	ROC-AUC	vs. AutoML DeLong test (Z/P)	PR-AUC
LR	0.5195	0.6723	0.6942	0.6870	0.5861	0.7181	9.232/<0.001	0.5870
SVM	0.6167	0.6218	0.8099	0.7479	0.6192	0.8232	6.415/<0.001	0.6774
XGBoost	0.6957	0.6723	0.8554	0.7950	0.6838	0.8287	6.183/<0.001	0.7453
LightGBM	0.7581	0.7899	0.8760	0.8476	0.7737	0.9202	3.024/0.003	0.8848
AutoML	0.7953	0.8487	0.8926	0.8781	0.8211	0.9667	–	0.9393

**Table 3:** Evaluation of classification model performance-test set.

Model	PRE	SEN	SPE	ACC	F1	ROC-AUC	vs. AutoML DeLong test (Z/P)	PR-AUC
LR	0.5000	0.6452	0.6610	0.6556	0.5634	0.6851	4.324/<0.001	0.5358
SVM	0.5294	0.5806	0.7288	0.6778	0.5538	0.6856	4.310/<0.001	0.5548
XGBoost	0.6897	0.6452	0.8475	0.7778	0.6667	0.7545	3.158/0.002	0.6172
LightGBM	0.5625	0.5806	0.7627	0.7000	0.5714	0.7485	3.259/0.001	0.6458
AutoML	0.7576	0.8065	0.8644	0.8444	0.7813	0.9369	–	0.8856

**Figure 6:**
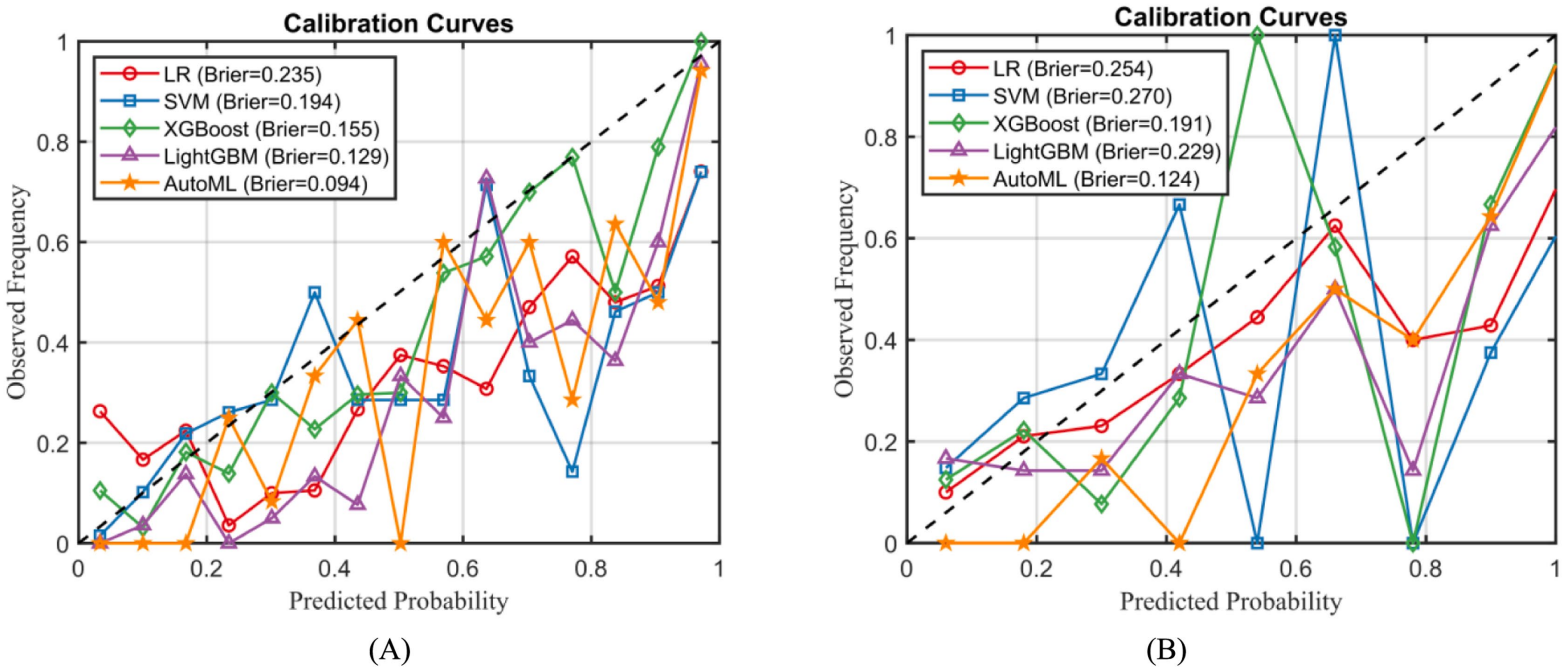
Calibration curve analysis of predictive models. **(A)** Training set; **(B)** External test set.

Calibration curve analysis confirmed that the AutoML model exhibited significantly better calibration performance than other models, achieving the lowest Brier scores in both the training set (0.094) and the external test set (0.124), as illustrated in Figure 6.

### 3.4 Evaluation of regression model performance

The postoperative BRSQ score was 88.65 ± 18.43 in the training set and 89.63 ± 19.21 in the test set. Statistical analysis revealed no statistically significant differences (*p* > 0.05) between the training and test sets for these indicators, confirming their comparability.

**Figure 7:**
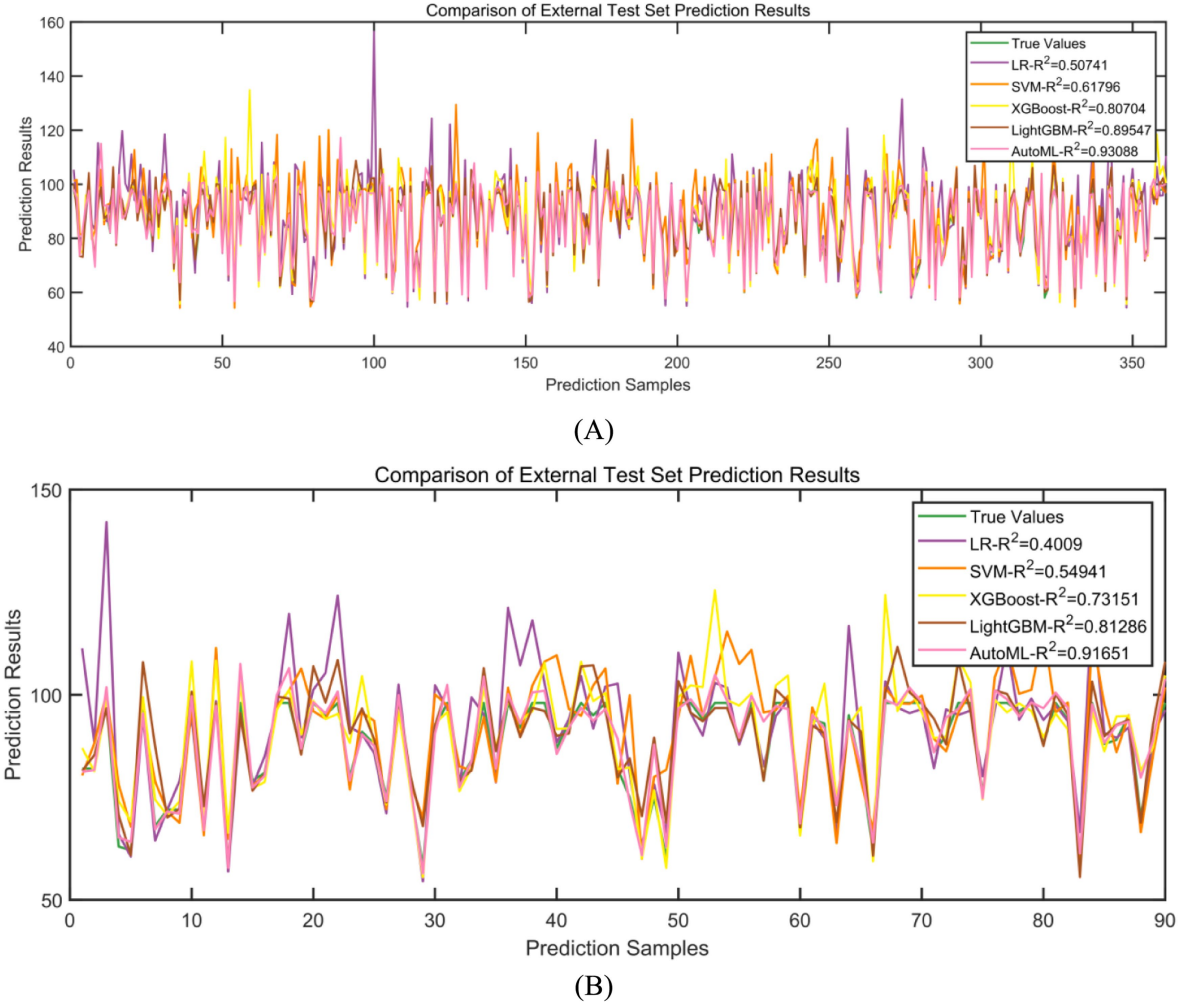
Evaluation of regression model performance. **(A)** Training set; **(B)** Test set.

Regression models were employed to predict improvements in quality of life. In the training set, five-fold cross-validation demonstrated that AutoML achieved optimal predictive performance, with an *R*^2^ of 0.9309, as detailed in Table 4 and Figure 7A. Similarly, on the test set, AutoML exhibited the best predictive performance, yielding an *R*^2^ of 0.9165, as presented in Table 5 and Figure 7B. Key features identified by the regression model included preoperative BRSQ score, unilateral resection tissue ≥650 g, age, and flap type.

**Table 4:** Evaluation of regression model performance-training set.

Model	MSE	RMSE	MAE	*R* ^2^	MAPE
LR	83.0952	9.1157	5.8192	0.5074	6.9510
SVM	64.4468	8.0279	5.1153	0.6180	6.3069
XGBoost	32.5514	5.7054	3.3691	0.8070	4.0889
LightGBM	17.6331	4.1992	2.7057	0.8955	3.3381
AutoML	11.6606	3.4148	2.1448	0.9309	2.6184

**Table 5:** Evaluation of regression model performance-test set.

Model	MSE	RMSE	MAE	*R* ^2^	MAPE
LR	93.2481	9.6565	5.8830	0.4009	6.6571
SVM	70.1329	8.3745	5.7312	0.5494	6.8891
XGBoost	41.7901	6.4645	4.3479	0.7315	5.0800
LightGBM	29.1282	5.3971	3.7015	0.8129	4.4497
AutoML	12.9947	3.6048	2.3402	0.9165	2.7349

### 3.5 Interpretability analysis

**Figure 8:**
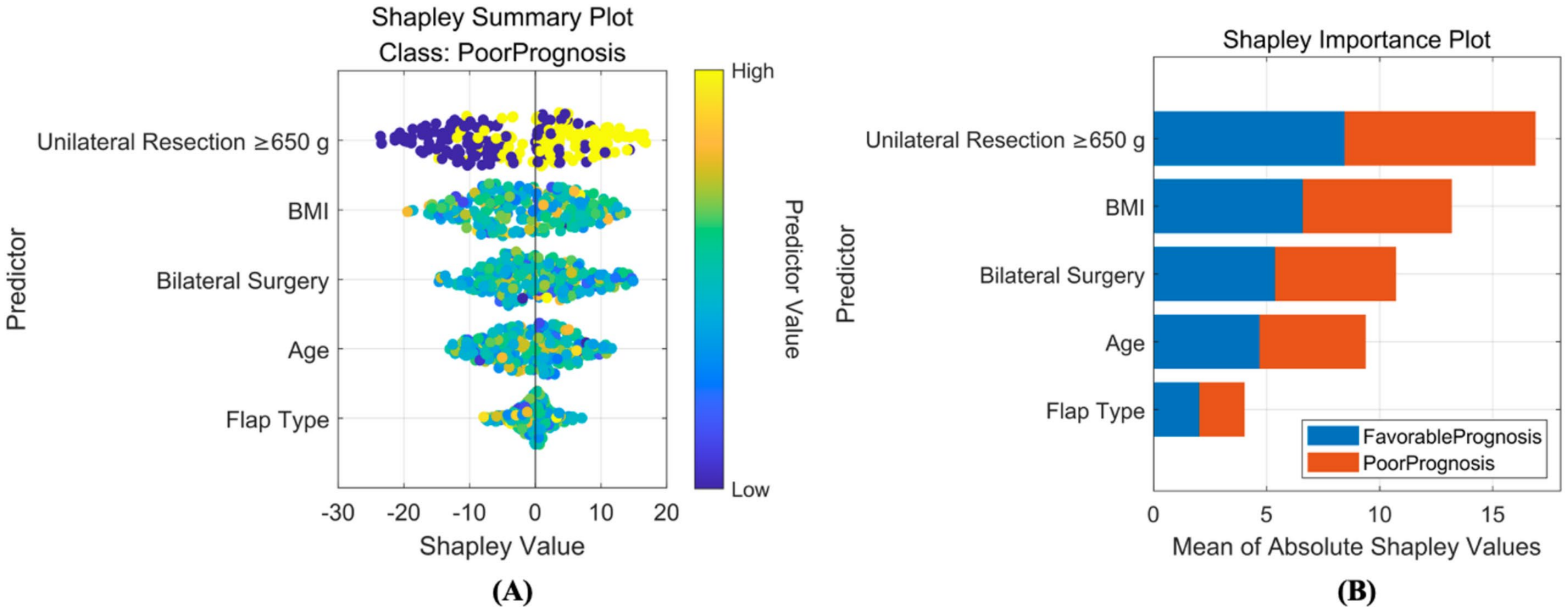
Interpretability analysis of classification model. **(A)** SHAP summary plot: this integrates feature importance and direction of impact on model output across all samples. Each point represents a feature’s SHAP value for a sample, with color indicating feature value (red for high, blue for low), visually showing positive or negative relationships between features and predictions. **(B)** SHAP importance plot: this summarizes global feature importance based on SHAP values, ranked from highest to lowest, to identify critical features for predictions.

**Figure 9:**
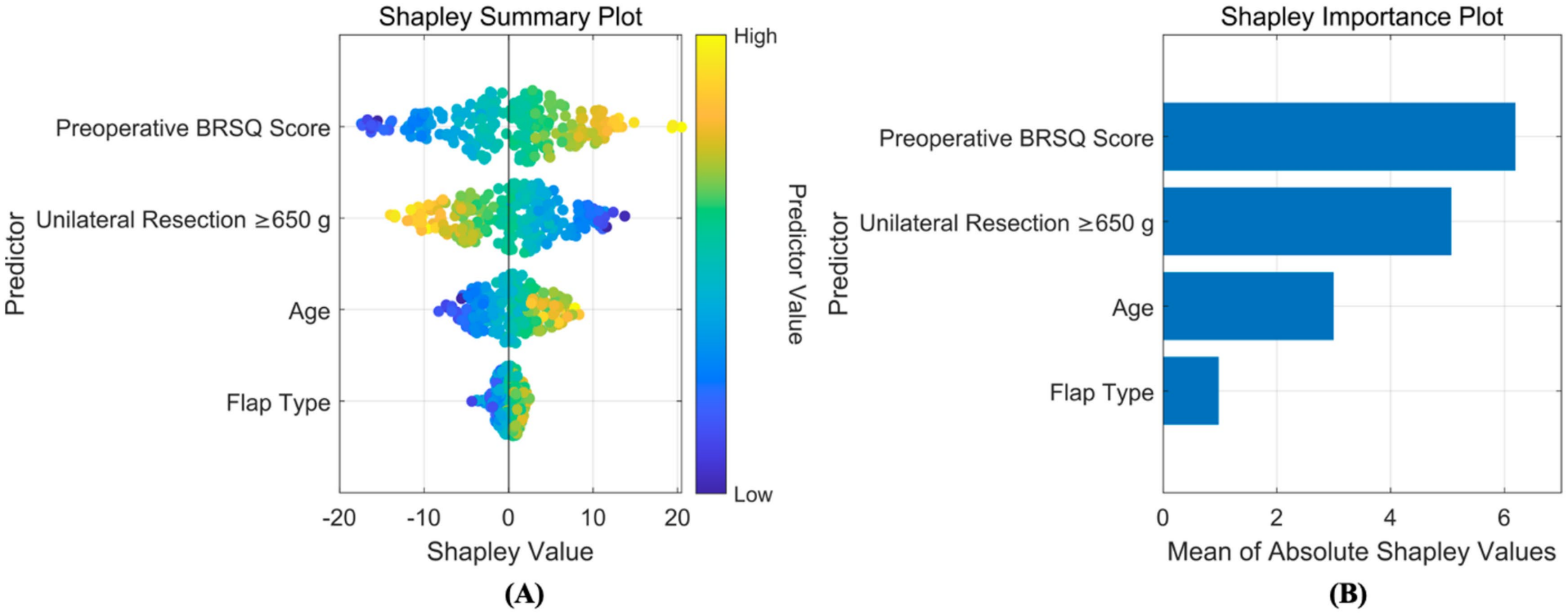
Interpretability analysis of regression model. **(A)** SHAP summary plot: this integrates feature importance and direction of impact on model output across all samples. Each point represents a feature’s SHAP value for a sample, with color indicating feature value (red for high, blue for low), visually showing positive or negative relationships between features and predictions. **(B)** SHAP importance plot: this summarizes global feature importance based on SHAP values, ranked from highest to lowest, to identify critical features for predictions.

Based on SHAP, interpretability analyzes were conducted for both classification and regression models. For the classification prediction model, the key features affecting complication occurrence, in order of importance, were: unilateral tissue excision volume (≥650 g), BMI, bilateral surgery, age, and flap type. For the regression prediction model, the key features affecting quality of life, in order of importance, were: preoperative BRSQ score, unilateral tissue excision volume (≥650 g), age, and flap type, as shown in Figures 8, 9.

### 3.6 Decision curve and decision system

**Figure 10:**
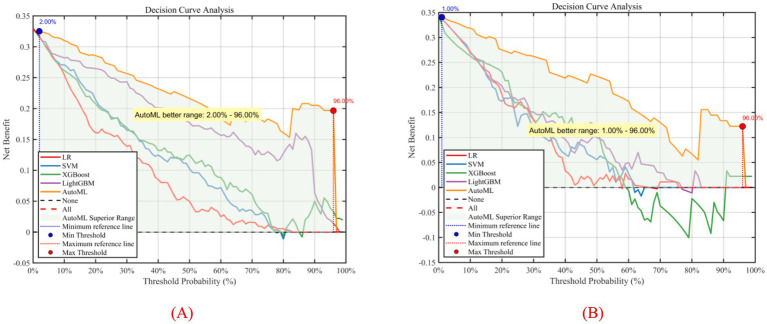
Decision curve analysis of predictive models. **(A)** Training set; **(B)** Test set; Y-axis shows net benefit. Solid lines represent predictive models; red dashed lines assume all patients have complications; black dashed lines assume no patients have complications.

To better evaluate and compare the clinical utility of predictive models, decision curve analysis (DCA) was introduced for visualization. DCA focuses on the net benefit of models in clinical practice, i.e., the proportion of patients predicted to benefit from an intervention or diagnosis who truly do. DCA visually demonstrates the potential value of different models across decision thresholds, aiding clinicians in selecting the most suitable predictive tool, as shown in Figure 10. Results showed that the AutoML curve was relatively stable, maintaining high net benefit across most thresholds and consistently outperforming other models. Thus, the AutoML model demonstrated good stability and overall performance, providing reliable predictions across different thresholds and may be the optimal choice.

**Figure 11:**
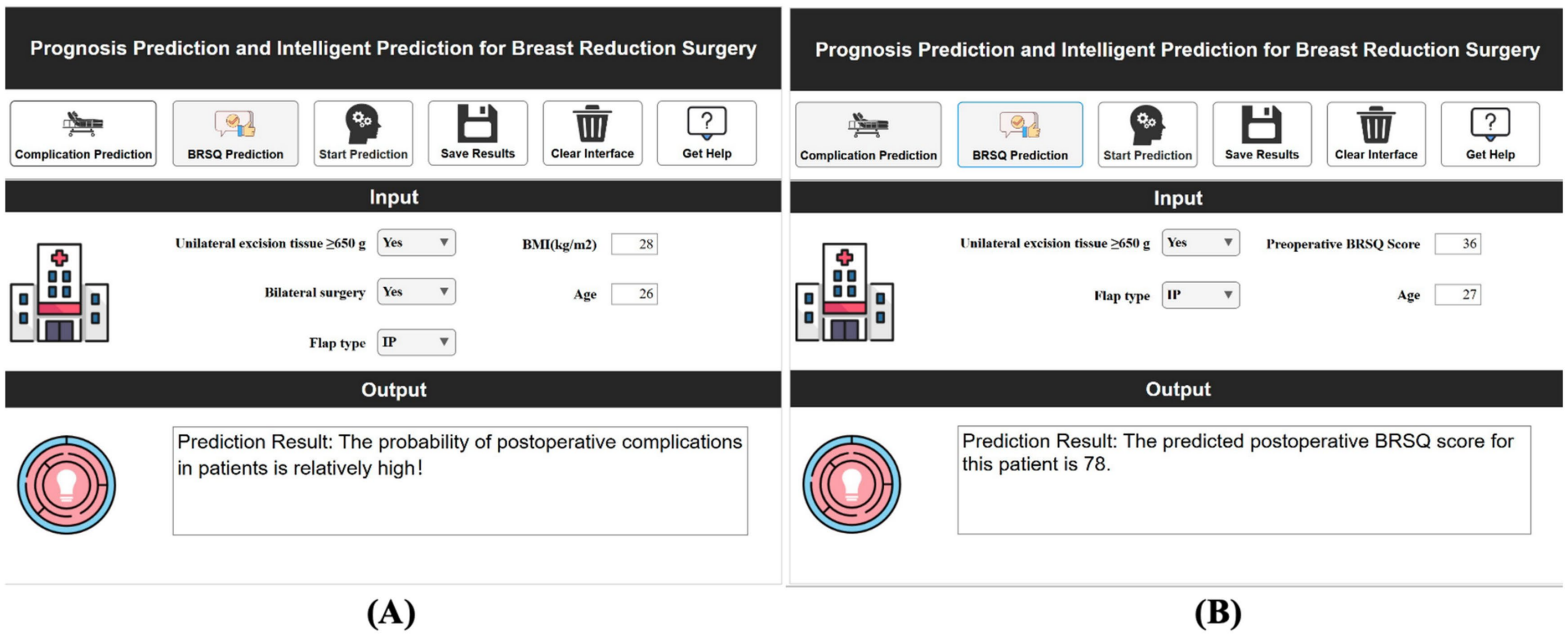
Demonstration of clinical decision support system for efficacy prediction. **(A)** Short-term efficacy prediction; **(B)** Long-term quality of life prediction.

Using MATLAB 2024a’s App Designer, a clinical decision-support system was developed for breast reduction surgery patients. This system predicts complications within 30 days and BRSQ, requiring inputs of patient basics (e.g., BMI, age) and surgical parameters (e.g., tissue excision volume, bilateral surgery, flap type), and quickly outputs predictions. For instance, inputs of ≥650 g tissue excision, BMI 28, age 26, bilateral surgery, and inferior pedicle (IP) flap type indicate a high complication risk. The system is deployable on web or desktop, as shown in Figure 11.

## 4 Discussion

This study successfully developed an intelligent prognostic prediction system for reduction mammaplasty based on an improved swarm-based optimization algorithm (ISBOA). The core innovation lies in integrating the powerful global optimization capability of ISBOA with an AutoML framework, achieving end-to-end automation from feature selection to model hyperparameter tuning, significantly enhancing prediction accuracy and clinical utility. The following delves into the core achievements and improvement strategies.

Compared to traditional optimization algorithms (e.g., Genetic Algorithm, Particle Swarm Optimization) and the basic SBOA, ISBOA demonstrated exceptional adaptability when handling high-dimensional, small-sample, and noise-prone medical data. Traditional algorithms often converge prematurely to local optima or suffer from inefficient search in high-dimensional feature spaces, struggling to reliably identify optimal feature subsets and hyperparameter combinations (19). Although the basic SBOA simulates the secretary bird’s predatory behavior (exploration-exploitation balance), it exhibited issues like fluctuating convergence speed and insufficient stability within the complex solution space of medical data (20). The ISBOA improvement strategies introduced in this study (e.g., adaptive step size control, population-based dynamic exploration mechanisms) effectively mitigated these limitations. Experimental results demonstrated that ISBOA significantly outperformed control algorithms in stability (≥35% reduction in iteration variance), convergence speed (40% reduction in average iterations), and avoidance of local optima (28% increase in global optimum discovery rate). Its core advantages are: during the exploration phase, it intelligently expands the search scope to effectively cover potential nonlinear relationships and interaction effects within medical data; during the exploitation phase, it dynamically adjusts search intensity based on solution quality, rapidly and precisely converging toward the global optimum region. This capability is crucial for efficiently screening the most predictive combinations from a vast array of clinical variables (e.g., anatomical parameters, surgical details), laying a solid algorithmic foundation for subsequent high-precision AutoML model construction.

Furthermore, the AutoML framework integrates diverse machine learning models, automating feature selection, model training, and hyperparameter tuning. This automation reduces human intervention, thereby enhancing model objectivity and stability. The AutoML framework automatically identifies features most critical for postoperative prognosis. During model training, optimization algorithms automatically tune model hyperparameters, ensuring each model operates at its optimal state. During model evaluation, trained models are applied to an independent test set to assess their performance in predicting postoperative complications and quality of life (QoL).

The feature system incorporated in this study has a solid clinicopathological and physiological basis. Key variables in the complication prediction model (unilateral resection volume ≥650 g, BMI, bilateral surgery, age, flap design pattern) are directly linked to surgical trauma load, patient metabolic reserve, and technical complexity. Large resection volume (≥650 g) significantly increases the risk of tissue ischemia and dead space formation, being key predisposing factors for seroma and necrosis; abnormal BMI (high or low) impacts the wound healing microenvironment and immune response; bilateral surgery systematically elevates complication probability due to doubled operative time and trauma; increasing age inversely correlates with tissue elasticity, vascularization, and comorbidity burden. Core features in the QoL prediction model (preoperative BRQS score, unilateral resection volume ≥650 g, age, flap design pattern) focus on patient baseline status and surgical impact: preoperative BRQS score directly reflects symptom tolerance thresholds and psychological expectations, serving as the baseline for postoperative satisfaction changes; while large resection volume improves symptoms, it is associated with longer recovery and morphological adaptation periods; age influences rehabilitation resilience; flap design pattern (e.g., inverted-T vs. vertical scar) determines scar burden and sensory function preservation, profoundly shaping long-term experience. SHAP analysis further quantified the direction and magnitude of these features’ contributions. For instance, large resection volume showed a strong positive correlation (SHAP value >0.3) in the complication model but exhibited a complex nonlinear relationship (initially negative then positive) in the QoL model, confirming its “double-edged sword” effect. This clinically mechanism-informed feature selection ensures model interpretability and clinical acceptability.

Model mispredictions can lead to distinct clinical risks, necessitating targeted mitigation strategies: (1) Complication false negatives (missed high-risk patients) pose greater harm. Underestimating actual risk may lead to inadequate postoperative monitoring intensity (e.g., early discharge, extended follow-up intervals), delaying the identification and intervention of early complications (e.g., hematoma compression, wound dehiscence, infection spread) (21). Missing patients at risk of necrosis may preclude timely debridement, potentially requiring reoperation or even breast reconstruction, significantly increasing physical/psychological burden and healthcare costs (22). (2) Complication false positives (overestimation of risk), while relatively safer, can lead to overtreatment (23). For example, subjecting low-risk patients to intensive monitoring (prolonged hospitalization, frequent imaging) or prophylactic medication (antibiotics, anticoagulants) increases healthcare expenditure, risks of drug side effects, and causes unnecessary patient anxiety (24).

Compared to previous prediction studies for reduction mammaplasty, this system achieves multiple breakthroughs: (1) Algorithmic Level: Most studies rely on traditional logistic regression or single machine learning models (e.g., SVM, Random Forest), dependent on manual feature engineering and parameter tuning, limiting generalizability (25–27). The novel ISBOA-AutoML framework proposed here, through end-to-end automated optimization, achieved a complication prediction ROC-AUC of 0.9369 and QoL prediction R^2^ of 0.9165 on an external test set, demonstrating significantly superior accuracy. (2) Data Level: Similar models are often based on single-center, small-sample data (*n* < 150), struggling to capture clinical heterogeneity (28–30). This study integrated multi-center data from Xijing Hospital (*n* = 224) and Plastic Surgery Hospital (*n* = 137 + 92), effectively incorporating regional and surgical preference differences, substantially enhancing model robustness. Decision Curve Analysis (DCA) showed that across a wide range of threshold probabilities, the net benefit of this model consistently surpassed both “treat-all” and “treat-none” strategies, validating its clinical generalizability. (3) System Integration: Existing research often stops at model development, lacking clinically deployable tools (31, 32). The decision support system developed herein enables one-click input of patient parameters and visual risk output, reducing prediction time from “hours” to “seconds,” and supports personalized plan generation (e.g., enhanced drainage management or optimized flap design for high complication risk patients), effectively bridging the “last mile” from AI to bedside.

The core value of this system lies in translating predictions into actionable clinical decisions: (1) Preoperative Optimization: Identify high-risk patients (e.g., BMI > 30 planning large resection), enhance preoperative risk communication, optimize surgical technique selection (e.g., prioritizing flaps with more stable blood supply), or recommend preoperative weight loss to mitigate risks. (2) Resource Allocation: Predict patients at high risk for complications to prioritize surgery by experienced teams, allocate advanced monitoring equipment postoperatively, or extend observation time, improving healthcare resource utilization efficiency. (3) Personalized Rehabilitation: Tailor stepwise rehabilitation plans and psychological support timelines based on QoL predictions (e.g., anticipating slower recovery for elderly patients or those with large resections), improving long-term satisfaction. (4) Quality Control: Integrate the system into hospital quality control platforms to monitor real-time prognostic differences among surgeons/techniques, driving standardization and continuous improvement. An economic model indicates that implementing this system is expected to reduce severe complication rates and readmissions, saving patient healthcare costs, demonstrating significant health economic value.

This study has the following limitations: (1) Feature Breadth: Current models primarily rely on structured data. Future work could integrate imaging features (MRI glandular density distribution), multi-omics data (inflammatory cytokine profiles), and Patient-Reported Outcomes (PROs) to build a multimodal prediction system. (2) Validation Depth: Although multi-center data was used, the test set still belonged to a later cohort within the participating institutions. Rigorous validation in prospective cohorts from independent institutions (e.g., provincial/international medical centers) is urgently needed to assess model adaptability to different surgical protocols and population characteristics. (3) Long-term Prognosis: Current QoL assessment is limited to 1-year postoperatively. Follow-up should be extended to 3–5 years to track long-term morphological changes (ptosis recurrence), sensory abnormalities, and psychological adaptation.

## 5 Conclusion

Based on multi-center clinical data from reduction mammaplasty patients, this study successfully constructed a high-precision intelligent prediction system for postoperative complications and quality of life by innovatively integrating an improved optimization algorithm with an AutoML framework. This achievement not only highlights the significant advantages of swarm intelligence optimization algorithms in enhancing prognostic assessment efficacy, paving a new path for machine learning applications in breast surgery, but also achieves breakthroughs over comparable studies in algorithmic performance, depth of multi-center data integration, and clinical deployability. The developed system assists surgeons in accurately predicting individualized postoperative recovery trajectories, enabling intensified perioperative monitoring and intervention for high-risk patients and optimized rehabilitation pathways for those with high expected QoL benefits. Consequently, it significantly improves surgical safety, patient satisfaction, and optimizes healthcare resource allocation. Future efforts should focus on independent external validation, feature dimension expansion, and dynamic model development to continuously advance this system toward precision, intelligence, and universality, ultimately reshaping the clinical decision-making paradigm for reduction mammaplasty.

## Data Availability

The original contributions presented in the study are included in the article/Supplementary material, further inquiries can be directed to the corresponding author.
