# Supplementary material for: Prognostic assessment and intelligent prediction system for breast reduction surgery using improved swarm intelligence optimization
Source: Front Med (Lausanne). 2025 Sep 11;12:1653201. doi: 10.3389/fmed.2025.1653201 (PMC12460295; doi:10.3389/fmed.2025.1653201)
Supplement: Supplementary file 1 [file Data_Sheet_1.DOCX]

| ISBOA伪代码 |
| --- |
| **function** [global_best] = **ISBOA**(dim, lb, ub, max_iter, objective_func)  % Phase 1: Sine Map Initialization  mu = 4.0; % Chaotic parameter  population_size = 100;  population = zeros(population_size, dim);    % Generate chaotic sequence  X_sine = rand(1); % Seed  **for** i = 1:population_size*dim  X_sine = (mu/4) * sin(pi * X_sine); % Sine map  population(i) = lb + (ub-lb)*(X_sine+1)/2; % Scaling  **end**  % Execute original SBOA framework  **for** iter = 1:max_iter  % Execute core SBOA search operators (hunting behavior modeling)  % (Original SBOA procedures applied here)    % Phase 2: Cauchy Mutation (Late-stage perturbation)  **if** iter > 0.7*max_iter  **for** i = 1:population_size  **if** rand() < 0.15 % Mutation probability  scale_factor = 0.5*(1 - iter/max_iter); % Adaptive γ  cauchy_noise = tan(pi*(rand()-0.5)); % C(0,1)  population(i,:) = global_best + scale_factor.*cauchy_noise;  % Boundary clipping  population(i,:) = max(min(population(i,:), ub), lb);  **end**  **end**  **end**  % Update global best solution  [fitness, global_best] = update_best(population, objective_func);  **end**  **end** |
