# Supplementary material for: Prognostic assessment and intelligent prediction system for breast reduction surgery using improved swarm intelligence optimization
Source: Front Med (Lausanne). 2025 Sep 11;12:1653201. doi: 10.3389/fmed.2025.1653201 (PMC12460295; doi:10.3389/fmed.2025.1653201)
Supplement: Supplementary file 2 [file Data_Sheet_2.DOCX]

**Table S1 Predictor Variable Definitions and Processing Standards**

| Variable Category | Variable Name | Variable Type | Unit/Definition | Missing Rate | Handling Method |
| --- | --- | --- | --- | --- | --- |
| Demographics | Age | Continuous | Years | <1% (Mandatory) | Listwise Deletion |
| Demographics | BMI | Continuous | kg/m² | <1% (Mandatory) | Listwise Deletion |
| Anatomy | N-SN Distance | Continuous | cm | 2.60% | Listwise Deletion |
| Surgery | Laterality | Categorical | Unilateral=0, Bilateral=1 | <1% (Mandatory) | Listwise Deletion |
| Surgery | Flap Type | Categorical | SMP=Superomedial, IP=Inferior, SP=Superior | <1% (Mandatory) | Listwise Deletion |
| Surgery | Resection Weight ≥650g | Binary | Yes=1, No=0 | <1% (Mandatory) | Listwise Deletion |
| Surgery | Operation Time | Continuous | Minutes | 2.20% | Listwise Deletion |
| Surgery | Postop Hospital Stay | Continuous | Days | 1.90% | Listwise Deletion |
| Comorbidity | Cardiovascular Disease | Binary | Present=1, Absent=0 | <1% (Mandatory) | Listwise Deletion |
| Comorbidity | Diabetes | Binary | Present=1, Absent=0 | <1% (Mandatory) | Listwise Deletion |
| Comorbidity | Smoking History | Binary | Present=1, Absent=0 | <1% (Mandatory) | Listwise Deletion |
| Preop Score | Preop BRSQ Score | Continuous | Points (Range:0-100) | 1.90% | Listwise Deletion |
| Outcome | Postop Complications | Binary | Hematoma/Infection/Dehiscence present=1 | 0% (Mandatory) | Not applicable (Complete) |
| Outcome | Postop BRQS Score | Continuous | Points (12±1 months postop) | 0% (Mandatory) | Not applicable (Complete) |
